# Supplementary material for: Piloting the Schistosomiasis Practical and Precision Assessment approach in five health districts of the N’zérékoré region, Republic of Guinea
Source: PLoS Negl Trop Dis. 2025 Oct 9;19(10):e0013413. doi: 10.1371/journal.pntd.0013413 (PMC12517497; doi:10.1371/journal.pntd.0013413)
Supplement: S1 Table — (DOCX) [file pntd.0013413.s001.docx]

**REPUBLIC OF GUINEA**

**FEASIBILITY EVALUATIONS**

S1 Table: Experience in determining practical assessments compared to precision assessments (Evaluation 1)

This form must be completed independently by the principal investigator and the co-investigators who were directly involved in the decision to start with practical evaluations for each district. Please complete this form once the decision has been made and before data collection has begun.

| Q1. | Please indicate the number of districts selected to begin the practical evaluation | - 5 districts selected for practical evaluation in Guinea |
| --- | --- | --- |
| Q2. | What information did you use to determine where to carry out the practical assessments? | - Baseline mapping data on CHS 2014 in Guinea and  - Coverage data for the various mass distributions 2016-2022 were used. |
| Q3. | Did you have to consult sources outside the SCH programs to obtain the information you needed to make this decision? If so, please describe the people or sources consulted and the information obtained. | - - The list of schools in Guinea was used - - The "ESPEN-Collect" platform was used to obtain the 2014 base mapping data and the coverage data for the various mass distributions for 2016-2022. - - The meeting of selected countries with the COR-NTD technical team to take account of the Guinean context. |
| Q4. | For each district, please indicate briefly the reason why the particular assessment (practical or precision) was chosen. | These 5 districts are the subject of a practical assessment for the following reasons:  - **Risk factors common to the CHS**: high rainfall with persistent bodies of water throughout the year, characterized by numerous marshes, rivers and swamps.  **- The activities of riverside populations are the same in these 5 SDs:** swamp rice cultivation and fishing are fairly well developed in these districts. This represents an important archetype in which it is necessary to understand how practical and precision assessment works.  - **The results of the baseline mapping (2011 to 2014)** showed that SCH prevalences were homogeneous with over 50% of the sites surveyed having a CHS prevalence above 50%.   - **- Every year since 2016,** these districts have benefited from mass treatment with Praziquantel, with coverage of over 75%. |
|  |  | in these 5 SDs: swamp rice cultivation and fishing are fairly well developed in these districts. This represents an important archetype in which it is necessary to understand how practical and precision assessment works.  - The results of the baseline mapping (2011 to 2014) showed that CHS prevalences were homogeneous with over 50% of the sites surveyed having a CHS prevalence above 50%.   - - Every year since 2016, these districts have benefited from mass treatment with Praziquantel, with coverage of over 75%. |
| Q5. | Is there any information you would have liked to have had access to that would have helped you make your decision? | Nothing to report |
| Q6. | Was the list of questions and recommended considerations in Table 2 helpful in making these decisions? Do you have any suggestions for improving the usefulness of this table? | The recommended considerations shown in Table 2 were very helpful in making the decision.  No further suggestions |
| Q7. | Are there any aspects of the practical and precision assessment approach that are unclear to you? | No, everything is clear |
| Q8. | What recommendations do you have for improving the orientation of national programs in order to determine whether it is appropriate to start with practical assessments or precision assessments in a given district? | No recommendation to make |
| Q9. | Is there anything else you'd like to add or recommend about your experience? | No recommendation to make |
