# Supplementary material for: Piloting the Schistosomiasis Practical and Precision Assessment approach in five health districts of the N’zérékoré region, Republic of Guinea
Source: PLoS Negl Trop Dis. 2025 Oct 9;19(10):e0013413. doi: 10.1371/journal.pntd.0013413 (PMC12517497; doi:10.1371/journal.pntd.0013413)
Supplement: S2 Table — (DOCX) [file pntd.0013413.s002.docx]

**REPUBLIC OF GUINEA**

**FEASIBILITY EVALUATIONS**

S2 Table: Feedback form on the implementation of practical evaluation (Evaluation 2)

Health district: N'Nzérékoré Team code 01

| Q1. | Please rank your experience by selecting the 15 PSU through systematic sampling for practical evaluation? | Very easy |
| --- | --- | --- |
| Q2. | If you found any aspect of the selection of the 15 PSU by systematic sampling difficult or confusing, please explain why. | Due to the lack of an updated database of schools available online, we were compelled to contact the Ministry of National Education to obtain the list of schools. |
| Q3. | Please rank your experience by selecting approximately 30 children at random from each PSU? | Very easy |
| Q4. | If you found any aspect of the children's selection difficult or confusing, please explain why. | Nothing to report |
| Q5. | How many days of training were needed for the field teams for the practical assessment? Was it sufficient? | We conducted three training sessions with the field teams according to the protocol; however, we believe that 4 days of training would be better. |
| Q6. | How many people made up each survey team and what were their roles? | Each team was made up of 4 people: two laboratory technicians, a supervisor/data entry operator, and a driver |
| Q7. | How many teams did you need to conduct the practical assessment in a single district? | 2 teams per health district. |
| Q8. | On average, how many hours did it take to carry out the sampling (of children) for the practical assessment in a single UPE (school)? | 30 – 45min |
| Q9. | Has a single survey team ever been able to conduct a sampling in two PSUs in one day? | Yes |
| Q10. | How many days (including travel days) did it take for the team or teams to conduct the practical assessment in a single district? | 8 days |
| Q11. | Which aspects, if any, of the fieldwork for the practical assessment were the most challenging for the survey teams? | The lack of electricity in rural areas, insufficient microscopes, obligations to read sample slides late into the night to finish. |
| Q12. | Which aspects, if any, of the fieldwork for the practical assessment were unclear and required clarification? | The procedure to follow if we have fewer than 32 samples per school in a school, the quantity of urine (10ml minimum). When a school in a sub-district is inaccessible without other replacement schools in the same sub-district, what course of action should be taken? |
| Q13. | Which parts of the fieldwork for the practical assessment were the easiest for the survey teams? | Administrative formalities and sampling of children in the age group concerned |
| Q14. | What recommendations do you have to improve the guidance or training materials for conducting practical assessments? | Provide the required number of microscopes, equipment, and supplies. |
| Q15. | Please describe who was involved in the data collection and management and how the process worked? | Nothing to report |
| Q16. | What worked well with the data management process? | Nothing to report |
| Q17. | What did not work well with the data management process? What recommendations do you have to improve it in the future? | Nothing to report |
| Q18 | Other comments | Nothing to report |

Health district: N'zérékoré Team code 02

| Q1. | Please rank your experience by selecting the 15 PSU through systematic sampling for practical evaluation? | Very easy |
| --- | --- | --- |
| Q2. | If you found any aspect of the selection of the 15 PSU by systematic sampling difficult or confusing, please explain why. | Due to the lack of an updated database of schools available online, we were prompted to contact the Ministry of National Education to obtain the list of schools. |
| Q3. | Please rank your experience by selecting approximately 30 children at random from each PSU? | Very easy |
| Q4. | If you found any aspect of the children's selection difficult or confusing, please explain why. | Determining the exact age of children in the absence of a reliable verification source; some may lie about their ages just to participate in the survey or not. |
| Q5. | How many days of training were needed for the field teams for the practical assessment? Was it sufficient? | We conducted three days of training with the field teams in accordance with the protocol; however, we believe that four days of training would be preferable. |
| Q6. | How many people made up each survey team and what were their roles? | Each team was composed of 4 people: two laboratory technicians, one supervisor/data entry operator, and one driver. |
| Q7. | How many teams did you need to conduct the practical assessment in a single district? | 2 teams per health district |
| Q8. | On average, how many hours did it take to carry out the sampling (of children) for the practical assessment in a single PSU (school)? | 30 – 45min |
| Q9. | Has a single survey team ever been able to conduct a sampling in two PSUs in one day? | Yes |
| Q10. | How many days (including travel days) did it take for the team or teams to conduct the practical assessment in a single district? | 8 days without catching up |
| Q11. | Which aspects, if any, of the fieldwork for the practical assessment were the most challenging for the survey teams? | The lack of electricity in rural areas, insufficient microscopes, and the obligation to read sample slides late into the night. |
| Q12. | Which aspects, if any, of the fieldwork for the practical assessment were unclear and required clarification? | The procedure to follow if we have fewer than 32 samples per school in a school, the quantity of urine (10ml minimum). When a school in a sub-district is inaccessible without other replacement schools in the same sub-district, what course of action should be taken? |
| Q13. | Which parts of the fieldwork for the practical assessment were the easiest for the survey teams? | Administrative formalities and sampling of children in the age group concerned |
| Q14. | What recommendations do you have to improve the guidance or training materials for conducting practical assessments? | Make available the required number of microscopes (two per team), materials, and inputs. |
| Q15 | Please describe who was involved in the data collection and management and how the process worked? | Data entry operators and supervisors |
| Q16 | What worked well with the data management process? | Assigning a unique code to each school and child, double entry of codes on forms and continuous data monitoring and feedback |
| Q17 | What did not work well with the data management process? What recommendations do you have to improve it in the future? | Nothing to report |
| Q18 | Other comments | Nothing to report |

Health district: Beyla Team code: 03

| Q1. | Please rank your experience by selecting the 15 PSU through systematic sampling for practical evaluation? | Very easy |
| --- | --- | --- |
| Q2. | If you found any aspect of the selection of the 15 PUS by systematic sampling difficult or confusing, please explain why. | Nothing to report |
| Q3. | Please rank your experience by selecting approximately 30 children at random from each PSU? | Very easy |
| Q4. | If you found any aspect of the children's selection difficult or confusing, please explain why. | Nothing to report |
| Q5. | How many days of training were needed for the field teams for the practical assessment? Was it sufficient? | Three days,  No |
| Q6. | How many people made up each survey team and what were their roles? | 5 people  A supervisor, a data entry operator, two laboratory assistants and a driver |
| Q7. | How many teams did you need to conduct the practical assessment in a single district? | Two teams |
| Q8. | On average, how many hours did it take to carry out the sampling (of children) for the practical assessment in a single UPE (school)? | 8h |
| Q9. | Has a single survey team ever been able to conduct a sampling in two UPEs in one day? | No |
| Q10. | How many days (including travel days) did it take for the team or teams to conduct the practical assessment in a single district? | 8 days, excluding catch-up |
| Q11. | Which aspects, if any, of the fieldwork for the practical assessment were the most challenging for the survey teams? | Material breakage (Cellophanes) |
| Q12. | Which aspects, if any, of the fieldwork for the practical assessment were unclear and required clarification? | Nothing to report |
| Q13. | Which parts of the fieldwork for the practical assessment were the easiest for the survey teams? | The mobilisation and involvement of the authorities |
| Q14. | What recommendations do you have to improve the guidance or training materials for conducting practical assessments? | - Ensuring proper planning of activities;  - Ensure that sufficient laboratory equipment and consumables are available before the teams leave for the field to avoid stock-outs and/or the recycling of certain materials;  - Allocate two days per site, including travel time between sites;  - Plan a day of advocacy and micro-planning before the start of the survey. |
| Q15. | Please describe who was involved in the data collection and management and how the process worked? | Nothing to report |
| Q16. | What worked well with the data management process? | Nothing to report |
| Q17. | What did not work well with the data management process? What recommendations do you have to improve it in the future? | Nothing to report |
| Q18 | Other comments | Nothing to report |

Health district: Beyla Team code: 04

| Q1. | Please rank your experience by selecting the 15 PSU through systematic sampling for practical evaluation? | Very easy |
| --- | --- | --- |
| Q2. | If you found any aspect of the selection of the 15 PUS by systematic sampling difficult or confusing, please explain why. | Nothing to report |
| Q3. | Please rank your experience by selecting approximately 30 children at random from each PSU? | Very easy |
| Q4. | If you found any aspect of the children's selection difficult or confusing, please explain why. | The special feature is that it is possible not to have all 30 SAC (10-14 year-olds) in a school |
| Q5. | How many days of training were needed for the field teams for the practical assessment? Was it sufficient? | No, an extra day, making a total of four days, would enable learners to better assimilate the training and anticipate all possible scenarios in the field. |
| Q6. | How many people made up each survey team and what were their roles? | Each team was made up of four people: two laboratory technicians responsible for taking samples, a supervisor in charge of the team, and a person responsible for data management. |
| Q7. | How many teams did you need to conduct the practical assessment in a single district? | Two teams would be enough to carry out such an activity in a district. |
| Q8. | On average, how many hours did it take to carry out the sampling (of children) for the practical assessment in a single UPE (school)? | 40 minutes on average, including administrative formalities. |
| Q9. | Has a single survey team ever been able to conduct a sampling in two UPEs in one day? | Yes |
| Q10. | How many days (including travel days) did it take for the team or teams to conduct the practical assessment in a single district? | On average, it took nine days for the two teams to carry out the survey in a single district. |
| Q11. | Which aspects, if any, of the fieldwork for the practical assessment were the most challenging for the survey teams? | Travel to the sites with the constant breakdown of vehicles coupled with the remoteness of certain sites as well as the inadequacy of certain inputs and equipment in particular (cellophane tape, microscope, etc.). |
| Q12. | Which aspects, if any, of the fieldwork for the practical assessment were unclear and required clarification? | A sample of less than 30 children.  The question was whether the 30 children could be supplemented by those from the community of the site in question. |
| Q13. | Which parts of the fieldwork for the practical assessment were the easiest for the survey teams? | -Community support  -Involvement of the authorities  -Sampling  -Reading slides |
| Q14. | What recommendations do you have to improve the guidance or training materials for conducting practical assessments? | - Increase the number of training days from three to four, i.e. two days of theoretical training and two days of practical training.  - Make sufficient equipment available for use  - Provide the teams with mobile logistics in good condition  - Always ensure that everything is ready before the start of activities |
| Q15. | Please describe who was involved in the data collection and management and how the process worked? | The supervisor in charge of the team was responsible for entering the data, although the whole team could do so depending on the internal organization set up by the team members. |
| Q16. | What worked well with the data management process? | - Monitoring the completeness and accuracy of the data submitted  - Encoding the forms to minimize data entry errors. |
| Q17. | What did not work well with the data management process? What recommendations do you have to improve it in the future? | Nothing to report |
| Q18 | Other comments | Nothing to report |

Health district: Yomou Team code: 5

| Q1. | Please rank your experience by selecting the 15 PSU through systematic sampling for practical evaluation? | Nothing to report |
| --- | --- | --- |
| Q2. | If you found any aspect of the selection of the 15 PUS by systematic sampling difficult or confusing, please explain why. | Nothing to report |
| Q3. | Please rank your experience by selecting approximately 30 children at random from each PSU? | Very easy |
| Q4. | If you found any aspect of the children's selection difficult or confusing, please explain why. | Nothing to report |
| Q5. | How many days of training were needed for the field teams for the practical assessment? Was it sufficient? | 3 days. Yes, that's enough |
| Q6. | How many people made up each survey team and what were their roles? | 4 people, 1 supervisor, 1 team leader and two biologists |
| Q7. | How many teams did you need to conduct the practical assessment in a single district? | 2 teams |
| Q8. | On average, how many hours did it take to carry out the sampling (of children) for the practical assessment in a single UPE (school)? | 3 hours |
| Q9. | Has a single survey team ever been able to conduct a sampling in two UPEs in one day? | No |
| Q10. | How many days (including travel days) did it take for the team or teams to conduct the practical assessment in a single district? | 9 days |
| Q11. | Which aspects, if any, of the fieldwork for the practical assessment were the most challenging for the survey teams? | Analysis of the samples |
| Q12. | Which aspects, if any, of the fieldwork for the practical assessment were unclear and required clarification? | Nothing to report |
| Q13. | Which parts of the fieldwork for the practical assessment were the easiest for the survey teams? | Collecting samples |
| Q14. | What recommendations do you have to improve the guidance or training materials for conducting practical assessments? | Add 1 day of work so that staff can master or become accustomed to analysis techniques. |
| Q15. | Please describe who was involved in the data collection and management and how the process worked? | For data management, the team leader rolls up the school headmaster and the children selected on the form. The biologists record the results in the register or bench notebook. After the analysis, the team leader retrieves the register to enter the data in the stool and urine results forms. |
| Q16. | What worked well with the data management process? | The use of electronic forms on tablets has worked well with the data management process |
| Q17. | What did not work well with the data management process? What recommendations do you have to improve it in the future? | The forms are linked together, and we are obliged to validate the first form before going on to the other. |
| Q18 | Other comments | Nothing to report |

Health district: YOMOU Team code 06

| Q1. | Please rank your experience by selecting the 15 PSU through systematic sampling for practical evaluation? | Nothing to report |
| --- | --- | --- |
| Q2. | If you found any aspect of the selection of the 15 PUS by systematic sampling difficult or confusing, please explain why. | Nothing to report |
| Q3. | Please rank your experience by selecting approximately 30 children at random from each PSU? | Very easy |
| Q4. | If you found any aspect of the children's selection difficult or confusing, please explain why. | Nothing to report |
| Q5. | How many days of training were needed for the field teams for the practical assessment? Was it sufficient? | 3 days but not enough |
| Q6. | How many people made up each survey team and what were their roles? | 4 people: 2 lab technicians for sampling, the supervisor and the data entry operator |
| Q7. | How many teams did you need to conduct the practical assessment in a single district? | Two for the 15 schools are sufficient |
| Q8. | On average, how many hours did it take to carry out the sampling (of children) for the practical assessment in a single UPE (school)? | 4 hours |
| Q9. | Has a single survey team ever been able to conduct a sampling in two UPEs in one day? | No |
| Q10. | How many days (including travel days) did it take for the team or teams to conduct the practical assessment in a single district? | 12 days including catch-up |
| Q11. | Which aspects, if any, of the fieldwork for the practical assessment were the most challenging for the survey teams? | If the children aren't properly motivated and aware, they won't take it seriously (especially the girls). |
| Q12. | Which aspects, if any, of the fieldwork for the practical assessment were unclear and required clarification? | What to do if a site does not have the required number of children. |
| Q13. | Which parts of the fieldwork for the practical assessment were the easiest for the survey teams? | Access to school management |
| Q14. | What recommendations do you have to improve the guidance or training materials for conducting practical assessments? | Increase availability of work materials and training time |
| Q15. | Please describe who was involved in the data collection and management and how the process worked? | The supervisor and data entry operator were involved in data entry and sent to the data manager. |
| Q16. | What worked well with the data management process? | The GPS system |
| Q17. | What did not work well with the data management process? What recommendations do you have to improve it in the future? | Sample preparation and slide reading given the time available |
| Q18 | Other comments | Nothing to report |

Health district: GUECKEDOU Team code 07

| Q1. | Please rank your experience by selecting the 15 PSU through systematic sampling for practical evaluation? | Nothing to report |
| --- | --- | --- |
| Q2. | If you found any aspect of the selection of the 15 PUS by systematic sampling difficult or confusing, please explain why. | Nothing to report |
| Q3. | Please rank your experience by selecting approximately 30 children at random from each PSU? | Very easy |
| Q4. | If you found any aspect of the children's selection difficult or confusing, please explain why. | Nothing to report |
| Q5. | How many days of training were needed for the field teams for the practical assessment? Was it sufficient? | Yes sufficient |
| Q6. | How many people made up each survey team and what were their roles? | 4 people  1 supervisor, team leader, data collector 2 laboratory technicians for sample analysis |
| Q7. | How many teams did you need to conduct the practical assessment in a single district? | We did it with 2 teams but we needed three (3) teams for the district. |
| Q8. | On average, how many hours did it take to carry out the sampling (of children) for the practical assessment in a single UPE (school)? | 5 hours |
| Q9. | Has a single survey team ever been able to conduct a sampling in two UPEs in one day? | Yes |
| Q10. | How many days (including travel days) did it take for the team or teams to conduct the practical assessment in a single district? | 10 days |
| Q11. | Which aspects, if any, of the fieldwork for the practical assessment were the most challenging for the survey teams? | Reading samples and accessibility in schools |
| Q12. | Which aspects, if any, of the fieldwork for the practical assessment were unclear and required clarification? | Nothing to report |
| Q13. | Which parts of the fieldwork for the practical assessment were the easiest for the survey teams? | Sampling children |
| Q14. | What recommendations do you have to improve the guidance or training materials for conducting practical assessments? | Adherence to training timetables.  - Increase the number of training days if necessary.  - Distribution of work kits and materials on time. |
| Q15. | Please describe who was involved in the data collection and management and how the process worked? | Works as part of a team |
| Q16. | What worked well with the data management process? | Nothing to report |
| Q17. | What did not work well with the data management process? What recommendations do you have to improve it in the future? | Nothing to report |
| Q18 | Other comments | Nothing to report |

Health district: Guéckédou Team code: 08

| Q1. | Please rank your experience by selecting the 15 PSU through systematic sampling for practical evaluation? | Nothing to report |
| --- | --- | --- |
| Q2. | If you found any aspect of the selection of the 15 PUS by systematic sampling difficult or confusing, please explain why. | Nothing to report |
| Q3. | Please rank your experience by selecting approximately 30 children at random from each PSU? | Very easy |
| Q4. | If you found any aspect of the children's selection difficult or confusing, please explain why. | Nothing to report |
| Q5. | How many days of training were needed for the field teams for the practical assessment? Was it sufficient? | 3 days of training. No, 3 days was not enough. |
| Q6. | How many people made up each survey team and what were their roles? | Each team was made up of 4 people:  1 supervisor who acted as team leader  1 data entry operator whose role was to coordinate the team.  2 laboratory technicians to collect and analyse the samples, interpret the results and record them in the register |
| Q7. | How many teams did you need to conduct the practical assessment in a single district? | 02 Teams |
| Q8. | On average, how many hours did it take to carry out the sampling (of children) for the practical assessment in a single UPE (school)? | 3 hours |
| Q9. | Has a single survey team ever been able to conduct a sampling in two UPEs in one day? | The team created one site per day. |
| Q10. | How many days (including travel days) did it take for the team or teams to conduct the practical assessment in a single district? | It took 9 days (not enough) |
| Q11. | Which aspects, if any, of the fieldwork for the practical assessment were the most challenging for the survey teams? | Collection and analysis on the same day |
| Q12. | Which aspects, if any, of the fieldwork for the practical assessment were unclear and required clarification? | Treatment of children who test positive |
| Q13. | Which parts of the fieldwork for the practical assessment were the easiest for the survey teams? | None of part was easy |
| Q14. | What recommendations do you have to improve the guidance or training materials for conducting practical assessments? | Consultancy for the purchase of equipment and consumables;  Increase the training time for interviewers from 3 to 4 days;  Allow one day before the start of the survey for meetings with the authorities and micro-planning at local level.  Allow 1.5 days per site and one day for inter-site travel.  Allow for rest days (Sundays) in the planning. |
| Q15. | Please describe who was involved in the data collection and management and how the process worked? | The supervisor acted as data entry operator.  - Collecting information from the site  - Enrolment of participants after they had presented two samples (stool and 10 ml urine) to the technicians  - Fill in the results on the Kato Katz and urine filtration forms after reading the slides. |
| Q16. | What worked well with the data management process? | The whole process |
| Q17. | What did not work well with the data management process? What recommendations do you have to improve it in the future? | Nothing to report |
| Q18 | Other comments | Nothing to report |

Health district: Lola Team code: 09

| Q1. | Please rank your experience by selecting the 15 PSU through systematic sampling for practical evaluation? | Nothing to report |
| --- | --- | --- |
| Q2. | If you found any aspect of the selection of the 15 PUS by systematic sampling difficult or confusing, please explain why. | Nothing to report |
| Q3. | Please rank your experience by selecting approximately 30 children at random from each PSU? | Very easy |
| Q4. | If you found any aspect of the children's selection difficult or confusing, please explain why. | No |
| Q5. | How many days of training were needed for the field teams for the practical assessment? Was it sufficient? | 3 days of training were insufficient |
| Q6. | How many people made up each survey team and what were their roles? | Each team consisted of 4 people:  1 supervisor who acted as team leader, 1 data entry operator whose role was to enter data into the telephones and register  2 laboratory technicians to collect and analyse the samples, interpret the results and record them in the register. |
| Q7. | How many teams did you need to conduct the practical assessment in a single district? | 2 teams |
| Q8. | On average, how many hours did it take to carry out the sampling (of children) for the practical assessment in a single UPE (school)? | 5 hours |
| Q9. | Has a single survey team ever been able to conduct a sampling in two UPEs in one day? | No |
| Q10. | How many days (including travel days) did it take for the team or teams to conduct the practical assessment in a single district? | 8 days (insufficient) |
| Q11. | Which aspects, if any, of the fieldwork for the practical assessment were the most challenging for the survey teams? | moving from one sub-district to another to meet the authorities, raise awareness, collect samples and analyse on the same day |
| Q12. | Which aspects, if any, of the fieldwork for the practical assessment were unclear and required clarification? | Nothing to report |
| Q13. | Which parts of the fieldwork for the practical assessment were the easiest for the survey teams? | Nothing to report |
| Q14. | What recommendations do you have to improve the guidance or training materials for conducting practical assessments? | Ensure that all inputs are available before the surveys;  Increase the number of training and survey days;  Plan a day to meet the prefectoral authorities.  Allow 1 day per site and one day for inter-site travel.  Take into account rest days (Sundays) in the planning. |
| Q15. | Please describe who was involved in the data collection and management and how the process worked? | Data management was carried out by the data entry operator supported by the team leader. Collecting information from the site  - Enrolment of participants after they had presented two samples (stool and 10 ml urine) to the technicians  - filling in the results on the Kato Katz and urine filtration forms after reading the slides. |
| Q16. | What worked well with the data management process? | The process went well overall |
| Q17. | What did not work well with the data management process? What recommendations do you have to improve it in the future? | Nothing to report |
| Q18 | Other comments | Nothing to report |

Health district: Lola Team code: 10

| Q1. | Please rank your experience by selecting the 15 PSU through systematic sampling for practical evaluation? |  |
| --- | --- | --- |
| Q2. | If you found any aspect of the selection of the 15 PUS by systematic sampling difficult or confusing, please explain why. |  |
| Q3. | Please rank your experience by selecting approximately 30 children at random from each PSU? | Very easy |
| Q4. | If you found any aspect of the children's selection difficult or confusing, please explain why. | Gender disparity in schools (more boys than girls in most schools);  Twinning or unification of two schools that were initially separated due to a lack of classrooms. |
| Q5. | How many days of training were needed for the field teams for the practical assessment? Was it sufficient? | 03  No, these 03 days were insufficient |
| Q6. | How many people made up each survey team and what were their roles? | 04  01 supervisor  01 data entry officer  01 senior laboratory technician (central level);  01 junior laboratory technician (operational level). |
| Q7. | How many teams did you need to conduct the practical assessment in a single district? | 02 teams of 04 people each. |
| Q8. | On average, how many hours did it take to carry out the sampling (of children) for the practical assessment in a single PUS (school)? | 02 hours. |
| Q9. | Has a single survey team ever been able to conduct a sampling in two PUSs in one day? | No, if of course we steal quality work because the schools are miles apart. |
| Q10. | How many days (including travel days) did it take for the team or teams to conduct the practical assessment in a single district? | Two (02) to three (03) days, 01 working day and 01 or 02 travel days |
| Q11. | Which aspects, if any, of the fieldwork for the practical assessment were the most challenging for the survey teams? | Access to sites with difficult access;  Generator failure; |
| Q12. | Which aspects, if any, of the fieldwork for the practical assessment were unclear and required clarification? | Working with or informing school headmasters; |
| Q13. | Which parts of the fieldwork for the practical assessment were the easiest for the survey teams? | Reading the slides |
| Q14. | What recommendations do you have to improve the guidance or training materials for conducting practical assessments? | Effective involvement of ECDs, school headmasters or teachers in the implementation process;  Providing extensive information and raising awareness before, during and after implementation; |
| Q15. | Please describe who was involved in the data collection and management and how the process worked? | Only the supervisor was in charge of data entry and management. Given the shortage of manpower during the sampling and slide preparation phases for the various samples, there are often delays in data entry, with the exception of the locality form, which is done before the others and does not require data entry. |
| Q16. | What worked well with the data management process? | Handling the various forms |
| Q17. | What did not work well with the data management process? What recommendations do you have to improve it in the future? | What didn't work well:  On-site verification of enrolment, kato katz and urine filtration data.  On-site entry of enrolment, kato katz and urine filtration data;  Timely reporting of enrolment, kato katz and urine filtration data.  The recommendations are as follows:  Separate the functions of supervisor and data entry agent.  Ensure that the supervisor and data entry agent have the right skills before making any choices;  Increase the number of team members;  Review the implementation period. |
| Q18 | Other comments | For future impact assessments, allow for travel time from the health districts to the sub-districts to be visited. |
