# Supplementary material for: Piloting the Schistosomiasis Practical and Precision Assessment approach in five health districts of the N’zérékoré region, Republic of Guinea
Source: PLoS Negl Trop Dis. 2025 Oct 9;19(10):e0013413. doi: 10.1371/journal.pntd.0013413 (PMC12517497; doi:10.1371/journal.pntd.0013413)
Supplement: S3 Table — (DOCX) [file pntd.0013413.s003.docx]

**REPUBLIC OF GUINEA**

**FEASIBILITY EVALUATIONS**

S3 Table: Programmatic Cost Form for a Practical Assessment Approach (Evaluation 3)

| Q1. | Did you find the practical evaluation affordable compared to previous mapping surveys on NTDs? | | 1 2 3 4 5  Abordable  Un litre d’eau | |
| --- | --- | --- | --- | --- |
| Q2. | If you find any aspect of the survey unaffordable, please explain why. | | Nothing to report | |
| Q3. | What was your unit cost for a litre /gallon of fuel? | | 12 000 GNF/Litre | |
| Q4. | What was the average fuel consumption per vehicle per day during the days of the trip? | | 86.49 L/ day / vehicle | |
| Q5. | What was the average fuel consumption per vehicle per day on the days the survey data were collected? | | 18.41 L/day/ vehicle | |
| Q6. | If you rented vehicles, (i) how many and (ii) what was the daily rental cost? | | 10 Vehicles in total GNF 1,000,000 per Day | |
| Q7. | For vehicles that were not leased, (i) how many and (ii) what were the maintenance costs of each for the entire survey? | | Nothing | |
| Q8. | What was the daily cost of hiring the training room? | | 700,000 GNF par jour | |
| Q9. | What was the cost of meals per person per day? | | GNF 90,000 per staff member per day | |
| Q10. Please list survey consumables and quantities purchased and quantities remaining at the end of the survey?  **This is not for audit purposes and is only to support the costing of these surveys to support future country guidance on consumables/equipment requirements.** | | | | |
| Consumables | | Quantity at start of survey | | Quantity at the end of the survey |
| Hydro-alcoholic gels (350 ml) | | 55 | | 25 |
| Bin liners (unit) | | 10 | | 0 |
| Paper towels (Roll) | | 80 | | 30 |
| Rubber wash glove (pair) | | 20 | | 10 |
| Examination gloves (50 pairs) | | 40 | | 4 |
| Surgical mask (50 pack) | | 30 | | 3 |
| Microscope slides Pack of 50 | | 150 | | 7 |
| Microscope slides 50 pack | | 150 | | 10 |
| 10CC syringes (pack of 100) | | 28 | | 2 |
| Urine cup with lid (unit) | | 2 400 | | 0 |
| Pipette Pasteur (10) | | 12 | | 2 |
| Urine disposal bucket(s) (Unit) | | 10 | | 0 |
| 1% hypochlorite solution (household bleach) (Unit) | | 10 | | 0 |
| Medicated soap (Unit) | | 40 | | 5 |
| Manual meter(s) (Unit) | | 10 | | 7 |
| Forceps (Unit) | | 12 | | 4 |
| Scissors (Unit) | | 10 | | 6 |
| Glycerol (litre) | | 10 | | 2 |
| Empty bottle (Unit) | | 10 | | 0 |
| Methyl alcohol (litre) | | 10 | | 6 |
| Waste container (Unit) | | 10 | | 0 |
| Laboratory coat (Unit) | | 260 | | 20 |
| Stool container (Unit) | | 2400 | | 0 |
| Labels (Roll) | | 120 | | 38 |
| Lugol (litre) | | 10 | | 1,5 |
| Register (Unit) | | 10 | | 0 |
| Rulers (Unit) | | 10 | | 0 |
| Note pads (Unit) | | 40 | | 0 |
| Pens (Unit) | | 40 | | 0 |
| Markers (Unit) | | 10 | | 0 |
| Binders (Unit) | | 40 | | 0 |
| Pencil (Unit) | | 10 | | 0 |
| Calculator (Unit) | | 10 | | 8 |
| Backpack (Unit) | | 10 | | 0 |
| Hematix box of 100 | | 30 | | 5 |
| Methylene blue (g) powder | | 9 | | 3 |
| Template (500) | | 6 | | 1 |
| Spatulas (500) | | 6 | | 1 |
| Journal | | 10 | | 0 |
| Metal sieve (500) | | 6 | | 1/2 |
| Telescope (unit) | | 20 | | 14 |
| Filter holder (Unit) | | 50 | | 28 |
| Cellophane (500) | | 12 | | 5 |
| Filter box of 100 | | 30 | | 1 |
