# Supplementary material for: Piloting the Schistosomiasis Practical and Precision Assessment approach in five health districts of the N’zérékoré region, Republic of Guinea
Source: PLoS Negl Trop Dis. 2025 Oct 9;19(10):e0013413. doi: 10.1371/journal.pntd.0013413 (PMC12517497; doi:10.1371/journal.pntd.0013413)
Supplement: S4 Table — (DOCX) [file pntd.0013413.s004.docx]

**REPUBLIC OF GUINEA**

**FEASIBILITY EVALUATIONS**

S4 Table: Interpretation Form for Practical and Precision Assessment Results (Evaluation 4)

| Q1. | How simple is the practical assessment decision tree (Figure 1) to understand? | Very easy |
| --- | --- | --- |
| Q2. | What aspects, if any, of the practical assessment decision tree did you find confusing or difficult to understand? | Nothing is confusing |
| Q3. | Once the practical assessment was complete, was your team able to interpret the results without outside help? If not, in what areas did you need help to interpret the results? | Yes, the team was able to interpret the results of the study |
| Q4. | Are you satisfied with the results of your practical assessment? Please explain. | The results of this assessment have given us a clear picture of the schistosomiasis situation in the region |
| Q5. | Has your practical assessment indicated that a precision assessment is required? If so, please describe what the SCH programme has decided to do in these contexts (e.g. will an accuracy assessment be conducted, if so, in how long? Will the programme use the results of the practical assessment to make treatment decisions at sub-district level without conducting an accuracy assessment? Etc.) | No, because the prevalence remains homogeneous and is above the 10% threshold in all the sites assessed; |
| Q5. | Will the SCH programme make any changes in response to the results of the practical evaluation? If so, please describe the changes. | Following the results of the survey, the programme modified its treatment strategy against schistosomiasis in the N'Zérékoré region by now including people aged over 15 in the treatment.  The Ministry plans to involve all administrative authorities at all levels more closely in raising awareness of the fight against faecal peril, by organising the national launch of the campaign in N'Zérékoré under the chairmanship of the Minister of Health himself. |
| Q6. | How likely are you to use this practical and accurate assessment approach in the future? | Very strong |
| Q7. | What additional (non-monetary) training or implementation resources would you  Would help you to carry out practical and detailed evaluations in the future? | Laboratory equipment: microscopes, cellophane paper, Kato Katz and urine filtration kits |
| Q8. | Do you recommend that changes be made to the practical and precise assessment approach? | We have not yet carried out a detailed precision evaluation survey to provide us with the comparative data we need to make an informed judgement. |
| Q9. | Would you like to comment further on the experience of implementing practical and accurate assessment? | Yes |
