# Supplementary material for: Piloting the Schistosomiasis Practical and Precision Assessment approach in five health districts of the N’zérékoré region, Republic of Guinea
Source: PLoS Negl Trop Dis. 2025 Oct 9;19(10):e0013413. doi: 10.1371/journal.pntd.0013413 (PMC12517497; doi:10.1371/journal.pntd.0013413)
Supplement: Attachment — Submitted filename: Response to Reviewers.docx [file pntd.0013413.s006.docx]

**Conakry le 29-04-2025**

**Editor-in-Chief PLOS Neglected Tropical Diseases 1875 Mission Street Suite 103 #188 San Francisco, CA 94103 USA**

**Subject: Responses to the reviewers regarding manuscript PNTD-D-24-01936**

Dear Editors and Reviewers,

We sincerely appreciate the insightful comments and constructive feedback provided during the review process for our manuscript, Piloting the Schistosomiasis Practical and Precision Assessment Approach in Five Health Districts of the N'zérékoré Region, Republic of Guinea. Your suggestions have greatly contributed to refining our study and strengthening the clarity and impact of our findings.

Below, we have systematically addressed each point raised by the reviewers:

1. **Methods**

Reviewer #1: Since the chi-square results were not significant, have the authors considered to use binomial logistic regression? Other suggestion would be to calculate (relative) risks.

*Thank you for this suggestion, however, we do not think that with performing different statistical tests and analyses that we will find a significant finding. Just looking at the data in Table 1, we can see (without a statistical test) that there does not appear to be a meaningful difference in SCH prevalence by age, sex, water use or fishing. Thus, we think the finding of non-significance via the chi-squared test is appropriate.*

Reviewer #2: However, it would be beneficial to clarify the reasons for excluding younger children from the study, as this could affect the evaluation of parasitic prevalence. While obtaining consent from the parents of younger students may be challenging, including this population could provide valuable information for a more comprehensive understanding of the studied population.

*We agree that infection in other age-groups is important and should be addressed through community-wide interventions. Age-infection studies highlight that although there is inter and intra-country variation, in general a higher prevalence of schistosome infection among children of school-going age around 10 to 14 years (*[*Senghor et al 2014*](https://doi.org/10.1186/1756-3305-7-5)*,* [*Angora et al 2019*](https://doi.org/10.3390/tropicalmed4030110)*,* [*WHO 2022*](https://www.who.int/publications/i/item/9789240041608)*,* [*Njikho et al 2023*](https://doi.org/10.3390/tropicalmed8120522)*). This is primarily due to behaviours that frequently expose them to contaminated water sources and potential acquired immunity in older children/adolescents (*[*Woolhouse 1998*](https://doi.org/10.1016/S0169-4758(98)01318-0)*,* [*Moira et al 2010*](https://doi.org/10.1371/journal.pntd.0000820)*,* [*Mitchell et al 2011*](https://doi.org/10.1371/journal.pcbi.1002237)*). Their participation is also valuable in facilitating the collection of samples (urine and stool), a process that is often challenging with younger children due to logistical constraints and the need for parental consent. Furthermore, a this age-group has been demonstrated as the ideal proxy for determining community-wide interventions (*[*Mwinzi et al 201*](https://doi.org/10.4269/ajtmh.15-0467)*5,* [*WHO 2022*](https://www.who.int/publications/i/item/9789240041608)*) This age group, having been significantly exposed to neglected tropical diseases, is therefore ideal for assessing the impact of mass drug administration treatments in schistosomiasis control programs, helping to guide therapeutic decisions at local and community levels.*

Additionally, the feasibility assessment is relevant and interesting. The researchers should present their results as they align with some of the study's objectives. If there are reasons why the results cannot be disclosed, the researchers should clarify and justify this decision.

*We are glad the reviewer finds the feasibility assessment to be relevant and interesting. We agree that understanding the programmatic feasibility of the tool is essential to determining if and how it should be scaled up. To this end, we have included, as an additional document, the responses to feasibility questions at various levels to facilitate better understanding.*

Reviewer #3: -Are the objectives of the study clearly articulated with a clear testable hypothesis stated?

Yes! The study aims to determine the current infection status and treatment strategy for each health sub-district and to evaluate the feasibility of the impact assessment approach. While the objectives are clearly articulated, a clear testable hypothesis is not explicitly stated. It would be beneficial to include a specific hypothesis to guide the study.

*Thank you for your comments. While this study was not designed as a hypothesis testing exercise, we do agree that it is important to state clearly the research hypothesis. Therefore, we have added the following statement to the main text (lines 122 - 124 in the revised version of the manuscript): The hypothesis going into this study is that the prevalence of schistosomiasis in school aged children will have decreased significantly following 5 to 8 rounds of preventive chemotherapy.*

-Is the study design appropriate to address the stated objectives?

The cross-sectional study design among children aged 10 to 14 years is appropriate for addressing the stated objectives. The systematic sampling of schools and random selection of children within schools ensures a representative sample. However;

- Practical assessment is the first step in the sampling process, requiring the random selection of schools across multiple sub-districts within an evaluation unit (district). The evaluation unit for this first step should be clearly defined as a health district. However, lines 139-141 seem to indicate that the subdistrict is the evaluation unit.

*Indeed, this is a mistake. This has been corrected to: The first stage used the Excel tool known as "Practical Assessment Systematic Sampling Tool" for the systematic random selection of 15 schools per health district, from a comprehensive list of schools within each health sub-district.*

- The definition of implementation unit within the context of Guinea should be correctly defined. Line 155-157 seems quite confusing. Current WHO advice is for countries to transition from district to subdistrict treatments. The latter should be the new implementation unit for MDA and evaluation unit for disease specific assessments. The context in Guinea should be clearly defined.

*Thank you for pointing that out, correction in lines 165 to 167 in the revised version of the manuscript: IU, or Implementation Unit, generally corresponds to a district, a county, a woreda, a circle, a health zone, a municipality, etc. In the context of Guinea, the IU refers to the health district. The sub-IU, a smaller administrative area within an Implementation Unit, represents the new unit for preventive chemotherapy against schistosomiasis. In the Guinean context, the sub-IU corresponds to the health sub-district.*

- How many teams were formed during data collection?

*For each health district, two teams, each consisting of four members, were deployed to carry out data collection.This has been made clearer in the text: Prior to data collection, two field teams each consisting of two experienced laboratory technicians, a data enumerator and a supervisor were trained for three days including one day of piloting.*

- Was the evaluation unit and sampling approach for SCH identical with STH? If so, highlight reason, if no, provide justification.

*Yes, the assessment of STH was performed opportunistically, integrated into the Practical Assessment survey. The Practical Assessment, which involves randomly sampling 20 students from each of 15 sites (totalling 300 students) within the IU, closely parallels the commonly used STH impact survey approach, where 5 sites with 50 students each (totalling 250 students) are sampled within each ecological zone or IU. However, compared with the often-used 5-site/50-student approach, the Practical Assessment exceeds WHO’s recommended sample size of 250 for moderate-to-heavy-intensity infection prevalence monitoring, enhances geographic representation, and reduces the margin of error around the IU-level prevalence estimate. Therefore, the Practical Assessment is comparably suited to guide STH treatment decisions and monitor progress toward elimination as a public health problem in the IU.*

2. **Results**

Reviewer #1: The result section was greatly presented. However, there was a mistake in Table 1, in which 1,537 should be written instead of 153 in number of positive Schistosomiasis. Moreover, Figure 2 was a bit blurry, the authors might want to consider enhancing the quality of the figure.

*Thank you for pointing that out, in Table 1 (now becomes Table 2 in the revised version of the manuscript), we corrected 153 to 1,537 for the number of positive schistosomiasis cases.*

*Thank you for pointing that out, the visual quality of Figure 2 has been enhanced.*

Reviewer #2: Additionally, the feasibility assessment is relevant and interesting. The researchers should present their results as they align with some of the study's objectives. If there are reasons why the results cannot be disclosed, the researchers should clarify and justify this decision.

*We are glad the reviewer finds the feasibility assessment to be relevant and interesting. We agree that understanding the programmatic feasibility of the tool is essential to determining if and how it should be scaled up. To this end, we have included, as an additional document, the responses to feasibility questions at various levels to facilitate better understanding.*

Reviewer #3: -Does the analysis presented match the analysis plan?
The analysis presented is concise though a plan wasn’t presented. However, the results are clearly and completely presented, with detailed tables and figures that enhance understanding.

*Yes, the analysis does align with the analytic plan included in the* [*Schistosomiasis Practical and Precision Manual*](https://espen.afro.who.int/tools-resources/advanced-analytical-tools/schistosomiasis-practical-precision-assessment-tool-sppa) *(v2) and the Guinea specific protocol.*

-Are the results clearly and completely presented?

The results are clearly presented, with comprehensive tables showing prevalence rates and confidence intervals. The figures are of high quality and provide clear visual representations of the data. However:

- The caption in Line 230 “Schistosomiasis prevalence by health district and health sub-district” doesn’t reflect the outputs. No details or highlights of subdistrict results have been provided

*Thank you for pointing that out. The Practical Assessment which was performed at the health district level and determines whether there is enough heterogeneity of prevalence to require a Precision Assessment or whether prevalence is homogeneously high (above >10%) and each of the health sub-districts should receive the same treatment strategy. We did not conduct a precision assessment (as the results showed the majority of sites were >10% prevalence), which would observed the prevalence results by sub-districts. Consequently, we have corrected the caption in line 230 (now located in line 261 in the revised version of the manuscript) to state: "Schistosomiasis prevalence by health district."*

- The SPPA method requires precision assessment upon completion of practical assessment. It should be clearly stated why precision assessment may not be required in this context. I presume it could be due to the homogenously high prevalence of Schisto.

*Correct, we have added the following language in lines 347 - 350: “Because all five health districts observed >8 schools exceeding the 10% prevalence threshold, a precision assessment was not warranted in any of the sites. The findings indicate that the health district remains the most appropriate implementation unit; it is not necessary to transition to a sub-IU treatment strategy at this time. “*

- Might be worth verifying if Lola health district qualifies for precision assessment.

*Although the Lola health district has one site with a prevalence of 6.3%, this is not sufficient to indicate heterogeneity. On the other hand, the 14 other sites have a prevalence above the threshold of 10%. This does not allow for a precision assessment as the decision lacks sufficient homogeneity.*

- A correlation between SCH infection intensity and micro/macro haematuria could be very useful in determining the extent of clinical morbidity associated with SCH.

*We agree with the reviewer that it is often useful to compare SCH infection intensity with micro and macro haematuria; however, because S. mansoni was the predominate SCH species in these five health districts, we believe this analysis would not be very insightful and best left for other settings where S. haematobium is more prevalent.*

-Are the figures (Tables, Images) of sufficient quality for clarity?

The tables are of sufficient quality for clarity. They effectively illustrate the prevalence of SCH-STH across the health districts surveyed. However, figures were cited but not provided, so it’s hard to conduct any appraisal of quality.

*The figures have been updated with a higher resolution and are provided for all reviewers.*

3. **Conclusions**

Reviewer #2: Another weakness in the choice of age group for schoolchildren was the methods used to determine the presence of parasites in feces. It should be noted that there are currently better tests for searching for parasites in feces, but they are not feasible in the reality of the study site. However, this could ideally contribute to determining more accurately the presence of parasites in feces.

*The reviewer raises a good point about more accurate (and resource intensive) tests for quantifying parasites in stool. An important aspect of this study was to assess the feasibility of the SPPA as it is likely to be implemented by national programmes. At present, the vast majority of national programmes are likely to continue using Kato-Katz to assess the prevalence of* S. mansoni *and STH in stool as it is a low cost approach for which technical capacity already exists. Hence our decision to stick with this diagnostic approach in the study.*

An essential aspect of the discussion lacking depth is considering external factors not assessed in the study. These factors, such as different age groups, health issues, medication availability, and resistance to antiparasitics, were mentioned but not explored. It would be beneficial to compare these factors with additional literature.

Reviewer #3: -Do the authors discuss how these data can be helpful to advance our understanding of the topic under study?

The authors discuss how these data can advance our understanding of schistosomiasis control and the effectiveness of mass drug administration (MDA) programs. However:

- No conclusions or recommendations were made with regards to continuity or pause of STH MDA based on the survey findings.

*Based on the recommendations of the WHO monitoring and evaluation framework for schistosomiasis and soil-transmitted helminthiases (https://www.who.int/publications/i/item/9789240099364) annexes, page 49, and following this impact assessment, the findings are as follows: i)- Beyla, Guéckédou, and Lola show a prevalence of STH infections ranging between ≥10% and <20%. Therefore, these areas will receive annual preventive chemotherapy for five years, followed by another impact assessment. ii)- Yomou has an STH prevalence between 2% and <10% and will receive preventive chemotherapy every two years, also followed by another impact assessment. iii)- N'Zérékoré, where the STH prevalence is below 2%, will see the suspension of preventive chemotherapy targeting entire at-risk groups. However, drug distribution will continue in appropriate settings, such as selected child-health visits, specific school years, or antenatal care visits.*

- PZQ resistance was cited as a possible reason for the persistent high SCH prevalence. Before making such presumption, it is worth asking the following questions:
  - 1.How often are coverage evaluation surveys conducted, and do these districts attain the min WHO threshold of 75% during post MDA coverage assessment?

*Thank you, we have added this text to line 350 : Unfortunately, no mass treatment coverage surveys for SCH and STH have been conducted in Guinea. The reported figures for mass treatment coverage are administrative data derived from health centre and Health District Directorate (DPS) reports following mass treatment campaigns”.*

- 2.What's the status of WASH infrastructure in the area?

*Although significant challenges remain in water and hygiene infrastructure in the N'Zérékoré region, the joint efforts of the Guinean government and its international partners demonstrate a commitment to addressing these issues. The modernization of existing facilities, the construction of new infrastructure, and the implementation of large-scale projects offer promising prospects for sustainably improving the living conditions of local populations.*

- 3.What are the behavioural risk exposures to SCH infection and what are the possible SBCC initiatives tailored to the context of N’zerekore Region?

*We included the most common behavioural risk exposures in our survey questionnaire and the results are shown in Table 2. Neither bathing or playing in water bodies the previous week nor fishing were found to be significantly associated with SCH infection.*

4. **Editorial and Data Presentation Modifications?**

Use this section for editorial suggestions as well as relatively minor modifications of existing data that would enhance clarity. If the only modifications needed are minor and/or editorial, you may wish to recommend “Minor Revision” or “Accept”.

Reviewer #1: Introduction

1. Line 66-67: please provide the correct citation and reference.

*References have been updated*

2. Line 69-71: please provide the correct citation and reference

*References have been updated*

3. Make sure to always write the full writings before using abbreviations

*Checked and corrected throughout*

Results

4. Table 1: please recheck and rewrite the correct data as this is very important

*Checked and corrected*

5. Line 243: the authors are subjected to only put the figure once as the figure was first mentioned in the manuscript

*Checked and corrected*

Reviewer #2: Minor Revision

Reviewer #3:

- Consider explicitly stating a clear testable hypothesis in the methods section to guide the study.

*See the response to this request above. A research hypothesis has been added (lines 122 - 124); however, we should emphasize that the goal of this study was not to conduct a hypothesis testing experiment but rather to pilot a new impact assessment approach.*

- Ensure consistency in the use of terms such as "school-age children" and "school-aged children."

*Thank you. These corrections have been made.*

- Minor grammatical corrections for improved readability, such as "school-age children" instead of "school-aged children."

*Thank you. These corrections have been made.*

- Sociodemographic information should be presented before the SCH and STH results.

*Thank you for this comment. The socio-demographic results have been moved to page 238-249 before those for SCH and STH. Thus the order of appearance of the tables has been updated in the various paragraphs of the manuscript where they were cited: the initial table 1 has become table 2 and the initial table 2 has become table 1.*
